# Simultaneous intracranial angiography and intraplaque hemorrhage imaging using SNAP

**DOI:** 10.1186/1532-429X-15-S1-E79

**Published:** 2013-01-30

**Authors:** Jinnan Wang, Xihai Zhao, Peter Boernert, Chun Yuan

**Affiliations:** 1 Philips Research North America, Briarcliff Manor, NY, USA; 2 BME, Tsinghua University, Beijing, China; 3 Philips Research Europe, Hamburg, Germany; 4 Radiology, University of Washington, Seattle, WA, USA

## Background

Intracranial atherosclerotic disease (IAD) accounts for 9-15% of all stroke incidents in the US [1], and the ratio is even higher in some racial groups [2]. Although angiography based imaging remains the prevalent diagnostic tool for IAD detection, it's unable to detect high risk lesions via direct visualization of the vessel wall. Lesions with intraplaque hemorrhage (IPH) on the carotid arteries have been associated with significantly increased clinical symptoms and plaque progress. An imaging tool that can detect both the luminal stenosis and high risk vessel wall disease is of clinical importance for IAD patient management. In this study, the recently proposed SNAP [3] technique was particularly optimized to simultaneously detect luminal stenosis and IPH for IAD patients.

## Methods

The SNAP technique was optimized toward the M1 segment of the middle cerebral arteries as it is the most frequent target of IADs. The optimized sequence has a shifted IR slab with a coverage of 25 cm.

One healthy volunteer and 3 patients with diagnosed IAD were recruited in this study. All MR scans were performed using a 3T whole body scanner (Philips Achieva, R3.21, the Netherlands) with an 8-ch brain coil. Geometrically matched SNAP and TOF scans were conducted on all subjects for easy comparison. For both scans, 1×1×1 mm^3^ isotropic resolution was acquired for a 160×160×50 mm^3^ FOV, the images were then zero-padded to 0.5×0.5×0.5 mm^3^ isotropic resolution. The SNAP images were reconstructed to allow MRA-only or MRA-IPH joint views, using a method described before [3].

## Results

**Figure 1:**
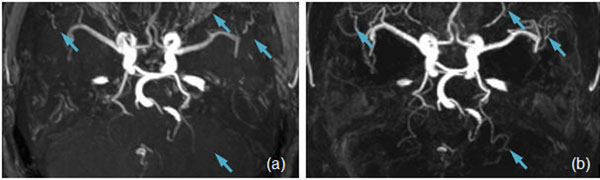
MRA images acquired on the same subject using TOF (a) and SNAP (b). Same spatial resolution and coverage were used. SNAP offers improved visualization of smaller arteries because of the improved background suppression (arrows).

The reconstructed 3D SNAP MRA provided improved visualization of the intracranial artery vascular tree, particularly on small branches, when compared to the matched TOF MRA images (Figure 1). The visualization of the major branches, such as MCA, is quite comparable between SNAP and TOF.

**Figure 2:**
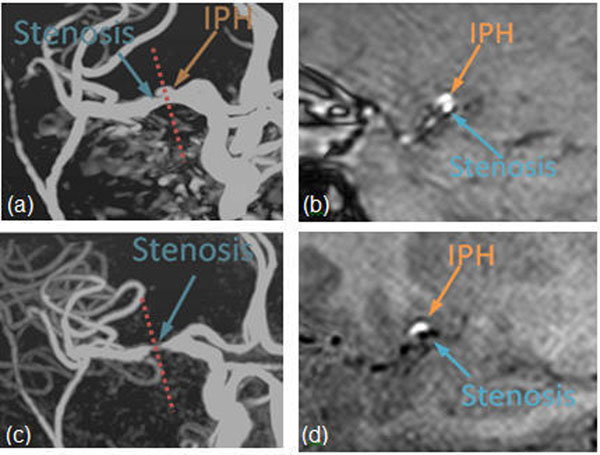
TOF (a) and Inverted-SNAP (c) images of a subject with suspected MCA stenosis and IPH. Both IPH and lumen were bright on TOF MRA (a), leading to a false-negative detection of both lesions; Inverted-SNAP MRA clearly visualized the stenosis (c). The cross-section image of both TOF (b) and SNAP (d) images confirmed the findings.

One patient was found to present stenosis and IPH on Inverted-SNAP but not on TOF (Figure 2a,c). Further examination revealed that no stenosis was identified on TOF MRA because the hyperintense IPH lesion caused false-negative findings. The cross-sectional TOF (Figure 2b) and SNAP (Figure 2d) images clearly confirmed the existence of both IPH and stenosis.

## Conclusions

The SNAP technique is for the first time optimized and applied to the intracranial artery atherosclerotic disease imaging. It promises to offer a unique approach to detect both luminal stenosis and high-risk intraplaque hemorrhage lesions for patients with IAD.

## Funding

NIH 1R01HL103609.

